# Doubling the Dose

**DOI:** 10.1371/journal.pbio.1001017

**Published:** 2011-02-08

**Authors:** William Mair

**Affiliations:** Freelance Science Writer, La Jolla, California, United States of America

**Figure pbio-1001017-g001:**
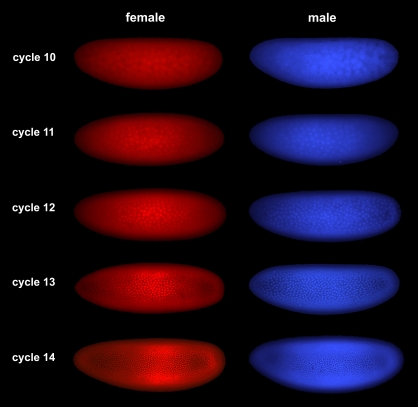
Sex-specific time course of early *Drosophila* development. Embryos at different stages were selected based on the number of nuclei they contain, and then genotyped to determine their sex.

[Fig pbio-1001017-g001]Maintaining the correct dosage of chromosomes is crucial for a cell to function properly. In sexually reproducing organisms most chromosomes exist in pairs, giving cells two copies of each of their genes, one inherited maternally and the other paternally. This is not always the case for the X/Y sex chromosomes found in many animals, however—although females have an X chromosome from each parent, males inherit a Y from their fathers in place of one X. The Y chromosome is a genetic wasteland that contains only a few functioning genes, meaning males are left with a half dose of an entire chromosome—the X. Such imbalance in gene dosage would be harmful were it not for the evolution of complex compensation mechanisms that restore sexual equality to the X chromosome. In this issue of *PLoS Biology*, Susan Lott and colleagues use new advances in sequencing to show for the first time that, not only does dosage compensation occur earlier in developing embryos than had previously been thought, but that it does so by a novel mechanism.

If an organism is to function correctly, the activity of its genes must be kept tightly regulated. This regulation can be compromised if part of a chromosome becomes deleted or duplicated, resulting in cells with either too many or too few copies of genes within that region. Changes to gene dosage can be disastrous for the cell. Indeed, spurious chromosomal rearrangements during fertilization are the cause of many genetic disorders such as such as Down's syndrome, whose sufferers have a duplication of some or all of chromosome 21. Animals have therefore evolved different strategies to prevent the differences in the sexes for X chromosome number being deleterious. In mammalian females, one copy of each X becomes densely packed and shut down, normalizing their X gene expression levels to the half dose of males, while in the nematode worm, both female X chromosomes are turned down half way. Fruit flies take the converse approach and hyper-activate the X genes in males such that they get a double dose. This hyper-activation is achieved by a group of proteins known as the “Male Specific Lethal” (MSL) complex, which bind specifically to the male X chromosome and uncoil the DNA, and in turn allow the genes to be turned up. Early on in developing embryos, however, not all the proteins needed for the MSL complex are present, meaning that there is a period during which the canonical dosage compensation machinery in inactive. Since regulation of gene expression is particularly critical in development, Lott and colleagues hypothesized that dosage compensation should also be crucial and that perhaps current techniques had failed to detect it.

Before they could look for compensation in early fly embryos, the scientists first had to develop new strategies to be able to test the expression levels of genes in a single embryo. During the initial stages of development, the embryo's genes are not yet active and the embryo relies on mRNA inherited from its mother in the egg to make proteins. After approximately ten cell divisions, the maternally inherited mRNAs begin to degrade and the embryo's own genome kicks in. There is a lag time, however, between the activation of embryonic genes and when the MSL dosage compensation complex is turned on. To test if dosage compensation of embryonic genes was occurring during this lag period, the scientists needed to distinguish the maternally inherited mRNAs from those expressed from the embryo's own DNA. They did this by studying offspring of parents from two genetically distinct populations, whose genomes they had first sequenced to identify DNA markers unique to each parent. Armed with these unique tags, the scientists could then determine if genes switched on in the embryos were maternal or embryonic, since maternal mRNA would have markers exclusively from the mother, while embryonic genes would contain a mix of both parental markers. Lott and colleagues adapted current sequencing methods to allow for the very low amounts of starting material available so that they could record all the genes that were active in a single fly embryo, and then categorize them as either maternal or embryonic.

When the researchers compared the expression levels of genes between male and female embryos during development, what they saw was striking—although the majority of genes were expressed at the same levels in males and females, genes on the X chromosome were much more active on the male X chromosome than either of the two female X chromosomes. Moreover, X genes with key roles in developmental showed up-regulation that resulted in perfect dosage compensation, even though males had half the dose of these genes compared to females the total activity level was the same in both sexes.

The finding that dosage compensation is occurring so early in development before the MSL complex is thought to be active raises new questions, as it suggests a novel compensation mechanism is at work. If the MSL complex is not responsible, dosage compensation may instead be controlled by a protein named SXL, which functions as the master regulator of sex determination in fruit flies, directing an embryo to develop into a female when switched on or a male when switched off. Alternatively, dosage control may be linked to the particular way chromosomes align. Just as the early dosage compensation is occurring, chromosomes become tightly associated with each other such that each gene is in close proximity to the second copy of itself. It may be that this association allows just one of each pair to turn on, while the other is inhibited. Since it has no partner to align with, the male X chromosome would remain switched on. Although more work is needed to test which of these hypotheses may be correct, the techniques developed in the current study give new resolution to the inner workings of gene expression in a developing embryo and how male flies compensate for their half dose of the X chromosome.

**Lott SE, Villalta JE, Schroth GP, Luo S, Tonkin LA, et al. (2011) Noncanonical Compensation of Zygotic X Transcription in Early *Drosophila melanogaster* Development Revealed through Single-Embryo RNA-Seq. doi:10.1371/journal.pbio.1000590**

